# Latin American Origin Is Not Associated with Worse Outcomes among Hospitalized Patients with COVID-19 in a Public Healthcare System

**DOI:** 10.3390/microorganisms9081772

**Published:** 2021-08-20

**Authors:** Silvia Otero-Rodriguez, Oscar Moreno-Pérez, Jose Manuel Ramos, Mar García, Vicente Boix, Sergio Reus, Diego Torrus, Pablo Chico-Sánchez, José Sánchez-Payá, Fernando Aldana-Macias, Joan Gil, Joaquín Portilla, Esperanza Merino, on behalf of COVID19 ALC Research Group

**Affiliations:** 1 Alicante Institute of Health and Biomedical Research (ISABIAL), 03010 Alicante, Spain; o.silvia.r@gmail.com (S.O.-R.); omorenoperez@hotmail.es (O.M.-P.); jose.ramosr@umh.es (J.M.R.); marinterna@gmail.com (M.G.); boix_vic@gva.es (V.B.); reus_ser@gva.es (S.R.); torrus_die@gva.es (D.T.); chico_pab@gva.es (P.C.-S.); sanchez_jos@gva.es (J.S.-P.); fercho306@hotmail.com (F.A.-M.); joangilcarbonell@gmail.com (J.G.); portilla_joa@gva.es (J.P.); 2 Unit of Infectious Diseases, Alicante General University Hospital, 03010 Alicante, Spain; 3 Endocrinology and Nutrition Department, Alicante General University Hospital, 03013 Alicante, Spain; 4 Clinical Medicine Department, Miguel Hernández University, 03202 Elche, Spain; 5 Internal Medicine Department, Alicante General University Hospital, 03010 Alicante, Spain; 6 Parasitology Area, Miguel Hernández University, 03202 Elche, Spain; 7 Preventive Department, Alicante General University Hospital, 03010 Alicante, Spain; 8 Pneumology Department, Alicante General University Hospital, 03010 Alicante, Spain

**Keywords:** COVID-19, origin, race, Latin American, mortality, outcome

## Abstract

Exploring differences in clinical outcomes based on race and origin among patients hospitalized for COVID-19 is a controversial issue. The ALC COVID-19 Registry includes all confirmed COVID-19 patients admitted to hospital from 3 March 2020 to 17 December 2020. The data were obtained from electronic health records in order to evaluate the differences in the clinical features and outcomes among European and Latin American patients. The follow-ups occurred after 156 days. A propensity score weighting (PSW) logistic regression model was used to estimate the odds ratio (OR, 95% CI) for Latin American origin and outcome associations. Of the 696 patients included, 46.7% were women, with a median age of 65 (IQR 53–67) years, 614 (88.2%) were European, and 82 (11.8%) were Latin American. Latin American patients were younger, with fewer comorbidities, and a higher incidence of extensive pneumonia. After adjusting for residual confounders, Latin American origin was not associated with an increased risk of death (PSW OR 0.85 (0.23–3.14)) or with the need for invasive mechanical ventilation (PSW OR 0.35 (0.12–1.03)). Latin American origin was associated with a shorter hospital stay, but without differences in how long the patient remained on mechanical ventilation. In a public healthcare system, the rates of death or mechanical ventilation in severe COVID-19 cases were found to be comparable between patients of European and Latin American origins.

## 1. Introduction

Several studies have suggested that COVID-19 infections could be more frequent among people of Latin American origin than among Europeans, and that both the need for hospital admission and instances of mortality would also be more frequent among them [1,2,3,4,5]. Deaths attributed to COVID-19 among Latin/Hispanic people in the USA are estimated to be 110.005 (18.4% of the total rate) [6]. Weighted population distributions, which more closely reflect differential risk within the areas and ages most affected by COVID-19, suggest that mortality may disproportionately affect those of Latin American origin [5]. In other registers, the relative risk of death was also higher (1.3–1.7 fold) among Latin Americans compared to Caucasians [1,4]. In large cities, such as Denver or New York, the proportion of deaths among Latin Americans almost doubled during the first months of the pandemic [7,8]. Finally, some European studies have also described more intensive care unit (ICU) admissions [9] or higher mortality rates among non-European people [2].

Whether these differences are due to biological factors, socioeconomic variables, or limited access to the healthcare system is not yet clear. The main criticisms of the available evidence are the lack of adjustment for confounders and that most of the studies have been carried out in North America.

The Spanish national healthcare system offers medical assistance to all people residing in Spain, including tourists, travelers, and even those who are in the country illegally, which minimizes inequity in access to health services. Spain has an estimated population of 47.4 inhabitants, 15.2% of whom were born abroad. There were 3.1 million Central and South American people living in Spain in 2021, representing 43.0% of the foreigners in Spain, and 6.5% percent of the entire population [10].

Understanding how the characteristics of patients differ between races/ethnicities, and which factors are associated with disease outcomes, is crucial for public health and for designing community-based interventions [4]. Unfortunately, such detailed characteristics remain sparse, and little is known regarding the relevance of racial/ethnic backgrounds in COVID-19 outcomes in Europe [11]. If the prognosis were actually worse based on patient ethnicity, it would be necessary to design specific protocols for this group of patients [3,4,12,13].

Our aim was to compare the clinical characteristics, complications, and outcomes among COVID-19 patients hospitalized in a public healthcare system according to their origin/race.

## 2. Materials and Methods

We conducted a retrospective cohort study of the 696 patients admitted for COVID-19 at the Alicante General University Hospital (ALC) (a tertiary Spanish national health service center) between 3 March and 17 December 2020.

### 2.1. Variables and Data Collection

Patient selection criteria: The ALC COVID-19 Data Registry includes all of the patients confirmed to be infected with SARS-CoV-2 who were hospitalized at ALC. The data registry is based on manual chart reviews and data extraction from electronic health records (EHR) conducted during the admission and until the end of the follow-up period after patients were discharged (12 February 2021). This also includes phone calls, in case of the absence of updated information in the patient’s EHR. Trained physicians collected data on several exploratory variables, including patient demographics, known SARS-CoV-2 epidemiologic risk factors, comorbid conditions, medications, COVID-19-related symptoms, laboratory tests, and patient outcomes. The main explanatory variable of the present analysis was Latin American origin.

### 2.2. Outcomes

The outcomes considered in this study include:

1. All-cause mortality (either in hospital or after discharge) and associated factors.
2. Invasive mechanical ventilation requirement and associated factors.
3. Secondary outcomes: The need for non-invasive ventilator support and ICU admission.

### 2.3. Statistics

The categorical and continuous variables are presented as frequencies (percentages) and medians (interquartile range, IQR), respectively. All tests were two-tailed, and a *p*-value of less than 0.05 was considered statistically significant. The final date of follow-up was 12 February 2021, unless censored.

The results presented below were stratified by self-identified origin, with statistical testing comparing the Latin and European groups via the Mann–Whitney U test (for numeric traits), the chi-squared test, and Fisher’s exact test (for binary outcomes), as appropriate. The propensity score weighting (PSW) was performed in a 1:1 ratio using nearest neighbor matching with a caliper width of 0.01, adjusted for prognostic variables, which differed between both sub-populations. Variables with losses greater than 15% were excluded from the adjustment.

The PSW multivariable logistic regression was fitted for outcomes; odds ratios (OR) with 95% confidence intervals (95% CI) were estimated for the association between race/origins and outcomes, and adjusted for covariates that persisted as potential confounding factors between the sub-populations after the PSW, with p-values below 0.05. The variables with losses greater than 15% were excluded. The WHO ordinary scale, which includes the outcomes evaluated, was not included in the regression models.

Finally, an adjusted lineal regression model was designed to evaluate the impact of race on the amount of time spent in the ICU or on mechanical ventilation.

IBM SPSS Statistics v25 (Armonk, NY, USA) was used for the analyses. The HGUA-ISABIAL ethics committee approved the study (expedient no. 200145); written informed consent was obtained from all the participants admitted as of 1 June. As the remaining patients were being included in a retrospective study, the need for informed consent from patients was waived.

## 3. Results

Of the 731 patients hospitalized in our center with COVID-19, the origin/race distribution was as follows: 84.0% (614) were European, 11.2% (82) were Latin American, 4.6% (34) were North African, and 0.14% (1) were from Sub-Saharan Africa. For this study, only the European and Latin American patients (N = 696) were included in the analysis.

The basal demographic characteristics, comorbidities, and clinical presentation by race/origins are shown in Table 1. The censored time was 156 (IQR 101–322) days for readmission, complications, or vital status.

### 3.1. Demographics and Comorbidities

As shown in Table 1, the Latin Americans were younger than the European patients, with a median age of 52 years (40–64) versus 67 (54–79), *p* < 0.001, without differences in gender (48.8% vs. 53.9% males). Comorbidities were less common among Latin Americans: with a lower median Charlson index (1 (0–2) vs. 3 (1–5), *p* ≤ 0.001), less hypertension (19.5% vs. 52.9%, *p* ≤ 0.001), and less diabetes (9.8% vs. 24.3%, *p* = 0.03). Other pathologies, such as chronic respiratory disease, immunosuppression, or obesity, did not achieve significant differences between the groups.

### 3.2. Clinical Presentation, Initial Assessment, and Treatment

The median times from symptoms beginning to hospitalization did not differ significantly between groups. The incidences of fever, respiratory symptoms, and systemic symptoms were similar in both groups. Similarly, differences in some acute phase reactants (C-reactive protein, procalcitonin, and ferritin) and lactate dehydrogenase levels did not achieve significance. While the median glomerular filtration rate was higher, and some prognostic markers (lymphocyte counts, Interleukin-6 (IL-6), D-dimer (DD), Troponin T (TnT), and Brain natriuretic peptide (BNP) levels) were more favorable among Latin Americans, there was a trend toward a higher frequency of extensive pneumonia (>50% of opacities in lung surface on X-rays) among them (33.3% vs. 23.5%, *p* = 0.09).

There were no differences among the main therapies used in both groups.

### 3.3. Main Outcomes and Associated Factors

The unadjusted mortality, need for ICU admission, and invasive ventilation support did not differ significantly between groups. All deaths happened while patients were still admitted to the hospital. The length of hospitalization, the number of days in the ICU, and the median number of days of invasive mechanical ventilation were also similar.

Basal demographic characteristics, comorbidities, and clinical presentation by race/origins after PSW are shown in Table 2.

As is shown in Table 2, the differences previously described between European and Latin American patients (Table 1) are minimized after matching. Demographic characteristics, including age and sex, were found to be comparable, as were comorbidities, with the exception of a higher percent of immunosuppression in the European group. Referring to the clinical presentations, initial assessment, and treatment, slightly higher levels of IL-6, TnT, and ferritin, along with an increased use of Tocilizumab, are evident in the European group.

**Figure 1:**
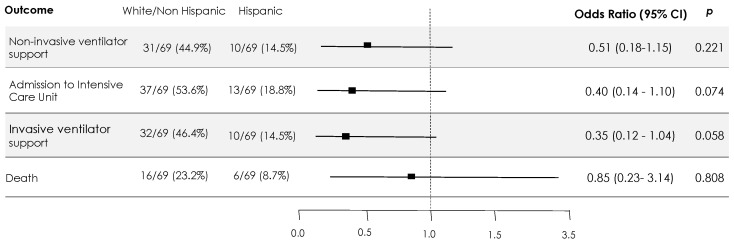
Main outcomes in a PSW multivariate analysis adjusted by residual confounding factors. Adjusted by immunosuppression, ferritin level, troponin level, asthenia in the first assessment, tocilizumab use. Interleukin 6 and PaO2:FiO2 were excluded, due to the lack of information about them in more than 15% of the patients. WHO ordinary scale, which includes the outcomes evaluated, was also excluded.

In the PSW multivariate analysis, which was adjusted by residual confounding factors, Latin American origin was not associated with more non-invasive ventilator support (PSW OR 0.51 (0.17–1.49)), ICU admissions (PSW OR 0.39 (0.14–1.09)), invasive mechanical ventilation requirements (PSW OR 0.35 (0.12–1.03)), or risk of death (PSW OR 0.85 (0.23–3.14)) (Figure 1). In turn, the adjusted linear regression analysis did not show that Latin American origin was associated with a longer time spent in ICU (B 3.195, *p* = 0.703) or on mechanical ventilation (B 6.089, *p* = 0.517). Nonetheless, hospital stays were shorter for the Latin American group (B −6.073, *p* = 0.039).

Other outcomes, such as pulmonary embolism (raw: 2.5% Latin American vs. 2.2% European, *p* = 0.698; after PSW: 2.5% Latin American vs. 3.1% European, *p* = 0.998) or hospital readmission (raw: 2.5% Latin American vs. 3.5% European, *p* = 0.842; after PSW: 2.5% Latin American vs. 1.6% European, *p* = 0.999), were also not influenced by patient origin.

## 4. Discussion

The present study did not find evidence of higher mortality rates or a greater need for invasive mechanical ventilation in Latin American patients hospitalized with COVID-19 in a public third-level hospital in Spain. Other important outcomes, such as ICU admission and requiring invasive mechanical ventilation, also did not differ between the groups, even in a PSW-adjusted multivariate model. Only the length of hospitalization was lower in the Latin American group. To our knowledge, this is the first study on hospitalized patients that addresses the effects of origin/race on the clinical course of COVID-19, adjusting in detail by a PSW model for the main known clinical confounding factors.

In ALC cohort, Latin patients were younger and with less comorbidities. They did not differ in the main inflammatory markers (C-reactive protein, ferritin, procalcitonin) except for IL-6, they had less lymphopenia and lower levels of BNP, DD, and TnT. Nevertheless, Latin patients showed a trend toward more frequent and extensive pneumonia.

The influence of Latin American origins in the prognosis of SARS-CoV-2 infections is controversial [13]. The CDC registers report that Latin Americans could be disproportionately represented among deaths attributed to COVID-19 [6] or have a more severe form of the illness [9], corroborating data from other studies in which COVID-19 fatalities in this sub-population could be around 1.5–2 times higher [1,4,7] than among Caucasians, although this finding is not consistent across all studies [14,15,16].

Selection bias, missing race/origins data, adjustment by confounding factors and key demographic covariates, inequalities in access to the health system, and incomplete outcome assessments in cohort and cross-sectional studies must be considered [13]. Two large studies, one population-based [1] and the other from a retrospective cohort [4], showed a significant adjusted difference in the hospitalization, deaths, ICU admissions, and the need for invasive mechanical ventilation, among Latin Americans compared to Caucasians. These studies highlighted age, gender, socioeconomic factors, and comorbidities as possible confounding factors that should be taken into consideration either jointly [1] or in separate models [4]. Prior to the pandemic, the city of Los Angeles reported lower rates of mortality among Latin American individuals compared to other ethnic groups; this situation reversed in 2020, with the rate of death among the Latin American population increasing to 714 per 100,000, while the death rate of non-Latin American people was only 699 per 100,000. COVID-19 was identified as the leading cause of death among Latin Americans [17]. Conversely, in other cohort studies, Ogedegbe et al. [14], Kabarriti et al. [15], and Rodriguez et al. [16] reported that the rates of adverse outcomes of COVID-19 did not differ by race/origin after adjustment for sociodemographic and clinical characteristics. Consistent with this evidence, Bassett et al. [18] showed that Hispanic/Latin American patients had fewer comorbidities, but comparable rates of ICU admission and death [18]. Globally, we can deduce that the higher crude mortality seen in Latin Americans is not generally confirmed after adjusting for COVID-19-cause-of-death. Instead of genetic factors, differences in virus exposure and healthcare access between ethnicities may underlie COVID-19-related disparities, influencing the outcomes [13]. The absence of differences in mortality from COVID-19 among Black, Hispanic/Latin American, and Caucasians in a large cohort of veterans with equal access to healthcare supports this hypothesis [19,20].

In Spain, which features a homogeneous national public healthcare system, Norman et al. [9] conducted a retrospective analysis of 2345 hospitalized patients with confirmed COVID-19 infections, and, after adjusting by race/gender, showed that there were no significant differences in mortality between Europeans and non-Europeans (mainly Latin Americans (91%))(OR 1.27, 95% CI: 0.86–1.88). However, an increase in ICU admission rates was found in non-Europeans (OR 1.43, 95% CI: 1.03–1.98). Recently, Díaz-Menéndez et al. [21], published a retrospective cohort study of 2226 hospitalized patients (486 migrants, 73.5% from South America) with confirmed SARS-CoV-2 infection; ICU admissions among the migrants, although higher than the general population, did not reach statistically significant differences. The multivariate analysis showed that being a migrant decreased the probability of dying (OR: 0.22; 95% CI: 0.15–0.33; *p* < 0.001) after adjusting for sex, age, and comorbidities [21]. A more detailed investigation of comorbidities and classical severity features not assessed in these retrospective studies may help to understand the complex relationship between higher ICU admission rates and the lack of a higher mortality rate.

We did not see any difference in the time between the onset of symptoms and arrival at the hospital, probably reflecting the universal access to the Spanish healthcare system. For those with more severe COVID-19-related symptoms, delayed entry into the healthcare system could have influenced the worsened outcomes in other series, and uncovered some of the social disparities affecting ethnic minorities [1,2,4,22].

Our study has limitations, but also illustrates several interesting points. It is a retrospective observational study, conducted with data from a single center, in a public health system, and with a limited sample size. We do not have data on certain epidemiological factors [3,4,12,13] (such as the number of cohabiting persons, patients’ current jobs or education level), which may impede the interpretation of the results. Data availability was limited to the electronic medical records at our institution. The use of PSW to minimize bias does not completely rule out the influence of some confounding factors, such as age and comorbidities, on our findings. Nevertheless, an outcome censored time greater than 5 months and the detailed adjustment for possible confounding factors using a PSW multivariate analysis reinforces and validates our findings.

## 5. Conclusions

Studying racial disparities and their possible influence on the prognosis of COVID-19 is important for understanding the nature of the disease, as well as for guiding public health policies and interventions. In our center, which is part of a public healthcare system, mortality, invasive mechanical ventilation requirement, non-invasive ventilator support, and ICU admission were not associated with Latin American origins. These findings are based on a single center with a small cohort of patients. Therefore, the presented data are still preliminary, and we look forward to further research confirming our results.

## Figures and Tables

**Table 1:** Basal demographic characteristics, comorbidities, and clinical presentation by origin/race.

	European [*n* = 614]	Latin American [*n* = 82]	*p*
**Demographics**			
Age, median (IQR), years	67 (54–79)	52 (40–64)	**<0.001**
Age ≥ 65 years old, % (N)	54.7% (336/614)	24.4% (20/82)	**<0.001**
Males, % (N)	53.9% (331/614)	48.8% (40/82)	0.382
Nosocomial, % (N)	5.5% (34/614)	1.2% (1/82)	0.109
Long-term care resident, % (N)	4.9% (30/614)	1.2% (1/82)	0.161
Health professional, % (N)	5% (31/614)	6.1% (5/82)	0.601
**Comorbidities**			
Hypertension, % (N)	52.9% (325/614)	19.5% (16/82)	**<0.001**
Diabetes, % (N)	24.3% (149/614)	9.8% (8/82)	**0.003**
Current or former smoker, % (N)	20.6% (70/339)	8.6% (5/58)	0.087
Obesity, % (N)	39.6% (168/424)	37.8% (17/45)	0.810
Chronic respiratory disease, % (N)	20.4% (125/612)	12.3% (10/81)	0.084
Immunosuppression, % (N)	5.7% (35/614)	2.5% (2/81)	0.298
Charlson comorbidity index, median (IQR)	3 (1–5)	1 (0–2)	**<0.001**
Charlson index ≥ 3, % (N)	56.67% (348/614)	17% (14/82)	<0.001
10-year expected survival ^†^	53.2 (0.9–90)	90.2 (0.95–95)	**0.016**
**Clinical Presentation**			
Median time (IQR) from symptom to hospitalization, days ^‡^	6 (3–9)	7 (4–9)	0.180
Fever, % (N)	70.4% (430/611)	70.7% (58/82)	0.947
Cough, % (N)	66.4% (405/610)	75.6% (62/82)	0.094
Dyspnea, % (N)	52.9% (324/612)	57.3% (47/82)	0.456
Diarrhea, % (N)	27% (164/607)	25.9% (21/81)	0.835
Confusion, % (N)	8.8% (54/560)	3.7% (3/82)	0.080
Fatigue, % (N)	44.2% (265/599)	37.5% (30/80)	0.253
Myalgias-arthralgias, % (N)	26.4% (159/603)	30%(24/80)	0.491
Anosmia-dysgeusia, % (N)	17% (102/599)	25% (20/80)	0.081
**Initial Assessment**			
WHO ordinal scale ^§^, % (N)			
4 5 6 7 8	70.0 (430) 8.6 (53) 1.5 (9) 5.0 (31) 14.9 (91)	76.8 (63) 6.1 (5) 0.0 (0) 9.8 (8) 7.3 (6)	**0.015**
Oximetry < 94% at room air, % (N)	45.8% (27/59)	33.8% (27/80)	0.151
PaO2:FiO2, median (IQR)	338 (281–404)	375 (300–416)	0.083
Respiratory rate, breaths/min, median (IQR)	16 (16–23)	17 (16–20)	0.813
Systolic BP, mmHg, median (IQR)	132 (116–146)	129 (116–143)	0.614
Diastolic BP, mmHg, median (IQR)	78 (67–89)	81 (70–89)	0.122
Temperature, °C, median (IQR)	36.9 (36.3–37.7)	36.7 (36.4–37.5)	0.339
Heart rate, beats/min, median (IQR)	92 (80–103)	94 (81–105)	0.221
eGFR, mL/min/m^2^, median (IQR)	78 (53–90)	90 (72–90)	**<0.001**
Lymphocytes, per mm^3^, median (IQR)	1000 (730–1380)	1200 (830–1560)	**0.011**
Lymphopenia, % (N)	49.1% (298/607)	34.6% (28/81)	**0.014**
C-reactive protein > 10 mg/dL, % (N)	172/605 (28.4)	25/82 (30.5)	0.699
Procalcitonin > 0.5 ng/mL, % (N)	10.5% (58/550)	8.0% (6/75)	0.495
Ferritin > 500 mg/L, % (N)	58.2% (331/569)	53.3% (40/75)	0.425
Lactate dehydrogenase > 250 U/L, % (N)	62.3% (335/538)	62.8% (49/78)	0.925
D-dimers > 1 mg/mL, % (N)	33.6% (184/548)	21.5% (17/79)	**0.03**
Interleukin 6 ≥ 10 pg/mL, % (N)	75.5% (349/462)	62.7% (42/67)	**0.025**
Troponin T > 14 ng/L, % (N)	39.1% (211/540)	9.3% (7/75)	**<0.001**
Brain natriuretic peptide > 125 pg/mL, % (N)	53.6% (288/537)	26.0% (19/73)	**<0.001**
Potassium mmol/L, median (IQR)	4 (3.7–4.4)	4 (3.8–4.3)	0.790
Opacities > 50% of lung surface on X-rays, % (N)	23.5% (112/476)	33.3% (21/63)	0.090
**Treatment**			
Antibiotic use for >48 h, % (N)	77.4% (261/337)	75.9% (44/58)	0.790
Corticosteroids, % (N)	61.9% (380/614)	67.1% (55/82)	0.362
Remdesivir, % (N)	9.5% (32/336)	6.9% (4/58)	0.521
Tocilizumab, % (N)	22.9% (139/606)	21% (17/81)	0.694

Data shown as %, median (interquartile range, IQR), unless specified otherwise. Statistically significant differences shown in bold. Percentages may not total 100 due to rounding. ^†^ 10-year expected survival derived from Charlson comorbidity index score. ^‡^ Days of symptoms before admission. OR: odds ratio; 95% CI: 95% confidence interval. ^§^ The categories are as follows: 0, non-infection; 1, not hospitalized and no limitations of activities; 2, not hospitalized, with limitation of activities, home oxygen requirement, or both; 3, hospitalized, not requiring supplemental oxygen and no longer requiring ongoing medical care (used if hospitalization was extended for infection-control or other nonmedical reasons); 4, hospitalized, requiring supplemental oxygen; 5, hospitalized, requiring noninvasive ventilation or use of high-flow oxygen devices; 6, hospitalized, receiving invasive mechanical ventilation or extracorporeal membrane oxygenation (ECMO); 7, hospitalized, receiving ventilation plus additional organ support–pressors, renal replacement therapy, or extracorporeal membrane oxygenation (ECMO); and 8, death.

**Table 2:** Basal demographic characteristics, comorbidities, and clinical presentation by origin/race after matching.

	European [*n* = 69 *]	Latin American [*n* = 69 *]	*p*
**Demographics**			
Age, median (IQR), years	59 (44–68)	54 (43–66)	0.178
Males, % (N)	62.3% (43/69)	53.6% (37/69)	0.301
Nosocomial, % (N)	1.4% (1/69)	1.4% (1/69)	1.000
Long-term care resident, % (N)	2.9% (2/69)	1.4% (1/69)	1.000
Health professional, % (N)	2.9% (2/69)	5.8% (4/69)	0.681
**Comorbidities**			
Hypertension, % (N)	31.9% (22/69)	18.8% (13/69)	0.078
Diabetes, % (N)	15.9% (11/69)	8.7% (6/69)	0.195
Current or former smoker, % (N)	25.7% (9/35)	9.8% (5/58)	0.062
Obesity, % (N)	34% (17/50)	34.3% (12/35)	0.978
Chronic respiratory disease, % (N)	17.4% (12/69)	14.7% (10/69)	0.669
Immunosuppression, % (N)	8.7% (6/69)	0.0% (0/69)	**0.028**
Charlson comorbidity index, median (IQR)	2 (0–3)	1 (0–2)	0.260
10-year expected survival ^†^	90 (0.9–97)	90 (0.9–96)	0.517
**Clinical Presentation**			
Median time (IQR) from symptom to hospitalization, days ^‡^	7.0 (4.5–8.5)	7.0 (4.5–9.5)	0.867
Fever, % (N)	81.2% (56/69)	72.5% (50/69)	0.226
Cough, % (N)	84.1% (58/69)	79.7% (55/69)	0.507
Dyspnea, % (N)	63.2% (43/69)	59.4% (41/69)	0.647
Diarrhea, % (N)	37.7% (26/69)	26.5% (18/68)	0.160
Confusion, % (N)	5.8% (4/69)	4.3% (3/69)	0.698
Fatigue, % (N)	55.9% (38/68)	38.2% (26/68)	**0.039**
Myalgias-arthralgias, % (N)	36.8% (25/68)	29.4% (20/68)	0.362
Anosmia-dysgeusia, % (N)	29.4% (20/68)	29.4% (20/68)	1.000
**Initial Assessment**			
WHO ordinal scale ^§^			
4, % (N) 5, % (N) 6, % (N) 7, % (N) 8, % (N)	31.9% (22/69) 11.6% (8/69) 10.1% (7/69) 23.2% (16/69) 23.2% (16/69)	79.7% (55/69) 4.3% (3/69) 0.0% (0/69) 7.2% (5/69) 8.7% (6/69)	**<0.001**
WHO ordinal scale > 4	68.1% (47/69)	20.3% (14/69)	**<0.001**
Oximetry < 94% at room air, % (N)	46.4% (32/69)	33.3% (23/69)	0.118
PaO2:FiO2, median (IQR)	338 (276–376)	383 (300–420)	**0.030**
Respiratory rate, breaths/min, median (IQR)	19 (16–25)	16 (16–20)	0.172
Systolic BP, mmHg, median (IQR)	128 (113–142)	130 (116–145)	0.474
Diastolic BP, mmHg, median (IQR)	80 (69–88)	80 (70–89)	0.564
Temperature, °C, median (IQR)	37.0 (36.4–37.8)	36.7 (36.4–37.5)	0.134
Heart rate, beats/min, median (IQR)	95 (87–103)	94 (81–105)	0.639
eGFR, ml/min/m^2^, median (IQR)	88 (70–90)	90 (75–90)	0.253
Lymphopenia % (N)	49.3% (34/69)	37.7% (26/69)	0.170
C-reactive protein > 10 mg/dL % (N)	34.8% (24/69)	29.0% (20/69)	0.465
Procalcitonin > 0.5 ng/mL, % (N)	9.0% (6/67)	7.7% (5/65)	0.793
Ferritin > 500 mg/L, %, (N)	79.1% (53/67)	58.5% (38/65)	**0.010**
Lactate dehydrogenase > 250 U/L, % (N)	76.5% (52/68)	64.7% (44/68)	0.132
D-dimers > 1 mg/mL, % (N)	27.5% (19/69)	21.7% (15/69)	0.429
Interleukin 6 ≥ 10 pg/mL, % (N)	79.2% (42/53)	37.9% (36/58)	**0.048**
Troponin T > 14 ng/L, % (N)	21.7% (15/69)	8.7% (6/69)	**0.033**
Brain natriuretic peptide > 125 pg/mL, % (N)	37.3% (25/67)	22.7% (15/66)	0.067
Potassium mmol/L, median (IQR)	4.2 (3.8–4.4)	4.0 (3.8–4.3)	0.212
Opacities > 50% of lung surface on X-rays, % (N)	40.6% (28/69)	47.8% (33/69)	0.391
**Treatment**			
Antibiotic use for >48 h	32/35 (91.4%)	39/51 (76.5%)	0.073
Corticosteroids	49/69 (71.0%)	47/69 (68.1%)	0.711
Remdesivir	0/35 (9.5%)	3/51 (5.9%)	0.267
Tocilizumab	36/69 (52.2%)	11/68 (16.2%)	**<0.001**

Data shown as %, median (interquartile range, IQR), unless specified otherwise. Statistically significant differences are shown in bold. Percentages may not total 100 due to rounding. * Losses of less than 10% of patients have been assumed, due to lack of information of some variables that differed between the two sub-populations (see Table 1), to ensure the accuracy of the PSW. ^†^ 10-year expected survival derived from Charlson comorbidity index score. ^‡^ Days of symptoms before admission. OR: odds ratio; 95% CI: 95% confidence interval. ^§^ The categories are as follows: 0, non-infection; 1, not hospitalized and no limitations of activities; 2, not hospitalized, with limitation of activities, home oxygen requirement, or both; 3, hospitalized, not requiring supplemental oxygen and no longer requiring ongoing medical care (used if hospitalization was extended for infection-control or other nonmedical reasons); 4, hospitalized, requiring supplemental oxygen; 5, hospitalized, requiring noninvasive ventilation or use of high-flow oxygen devices; 6, hospitalized, receiving invasive mechanical ventilation or extracorporeal membrane oxygenation (ECMO); 7, hospitalized, receiving ventilation plus additional organ support–pressors, renal replacement therapy, or extracorporeal membrane oxygenation (ECMO); and 8, death. Matching variables which differed between both sub-populations in Table 2, with losses greater than 15% excluded from the PSW logistic regression model: interleukin 6, brain natriuretic peptide.

## Data Availability

Access to data: S.O.-R., O.M.-P., and E.M. have full access and are the guarantors for the data, which is available on request.
